# Genetic Architecture of Variation in the Lateral Line Sensory System of Threespine Sticklebacks

**DOI:** 10.1534/g3.112.003079

**Published:** 2012-09-01

**Authors:** Abigail R. Wark, Margaret G. Mills, Lam-Ha Dang, Yingguang Frank Chan, Felicity C. Jones, Shannon D. Brady, Devin M. Absher, Jane Grimwood, Jeremy Schmutz, Richard M. Myers, David M. Kingsley, Catherine L. Peichel

**Affiliations:** * Division of Human Biology, Fred Hutchinson Cancer Research Center, Seattle, Washington 98109; † Graduate Program in Neurobiology and Behavior, University of Washington, Seattle, Washington 98195; ‡ Graduate Program in Molecular and Cellular Biology, University of Washington, Seattle, Washington 98195; § Department of Developmental Biology and Howard Hughes Medical Institute, Stanford University, Stanford, California 94305, and; ** HudsonAlpha Institute for Biotechnology, Huntsville, Alabama 35806

**Keywords:** sensory system, evolution, lateral line, lateral plates, QTL mapping

## Abstract

Vertebrate sensory systems have evolved remarkable diversity, but little is known about the underlying genetic mechanisms. The lateral line sensory system of aquatic vertebrates is a promising model for genetic investigations of sensory evolution because there is extensive variation within and between species, and this variation is easily quantified. In the present study, we compare the lateral line sensory system of threespine sticklebacks (*Gasterosteus aculeatus*) from an ancestral marine and a derived benthic lake population. We show that lab-raised individuals from these populations display differences in sensory neuromast number, neuromast patterning, and groove morphology. Using genetic linkage mapping, we identify regions of the genome that influence different aspects of lateral line morphology. Distinct loci independently affect neuromast number on different body regions, suggesting that a modular genetic structure underlies the evolution of peripheral receptor number in this sensory system. Pleiotropy and/or tight linkage are also important, as we identify a region on linkage group 21 that affects multiple aspects of lateral line morphology. Finally, we detect epistasis between a locus on linkage group 4 and a locus on linkage group 21; interactions between these loci contribute to variation in neuromast pattern. Our results reveal a complex genetic architecture underlying the evolution of the stickleback lateral line sensory system. This study further uncovers a genetic relationship between sensory morphology and non-neural traits (bony lateral plates), creating an opportunity to investigate morphological constraints on sensory evolution in a vertebrate model system.

A major challenge of evolutionary genetics is to identify the specific genetic changes that mediate variation in adaptive phenotypes. Sensory systems play a particularly important role in allowing animals to locate mates, acquire food, and avoid predators; thus, changes in sensory perception can have profound evolutionary consequences (Endler 1992). Modification of peripheral sensory receptors is predicted to be a major mechanism of change in perceptual capabilities (Wilczynski 1984). In support of this hypothesis, differences in peripheral sensory receptors have been correlated with perceptual differences in several vertebrate species (Yokoyama and Yokoyama 1996; Keller *et al.* 2007; Yoshizawa *et al.* 2010). In the visual system, *de novo* modifications to sensory receptors are sufficient to enable novel perceptual experiences in mice (Jacobs *et al.* 2007). However, with the exception of the visual system (Jacobs *et al.* 1996; Hofmann *et al.* 2009; Carleton *et al.* 2010), the genetic mechanisms that underlie variation in peripheral sensory receptors have not been identified in natural vertebrate populations.

The lateral line system is a promising model to investigate the genetic changes that underlie sensory system evolution. This mechanoreceptive sensory system enables fish and amphibians to sense water flow in aquatic environments (Dijkgraaf 1963; Bleckmann 1993). The peripheral receptors of the lateral line are neuromasts, which are bundles of hair cells distributed across the body surface, and they are readily observable using vital dyes. The lateral line plays an important role in a number of behaviors, including schooling (Pitcher *et al.* 1976; Pitcher 1979; Partridge and Pitcher 1980), prey localization (Montgomery and Macdonald 1987; Bleckmann and Bullock 1989; Montgomery 1989; Janssen *et al.* 1999; Yoshizawa *et al.* 2010), and rheotaxis (Montgomery *et al.* 1997; Baker and Montgomery 1999). Recently, the lateral line has become a model system for developmental genetics, resulting in the identification of genes and signaling pathways involved in neuromast development and patterning (Dambly-Chaudiere *et al.* 2003; Ghysen and Dambly-Chaudiere 2004; Ma and Raible 2009). This literature provides a rich resource to identify and functionally test the contribution of specific candidate genes to the evolution of the lateral line system.

Lateral line patterning varies both within and between species of fish, with differences observed in both the number and distribution of neuromasts (Dijkgraaf 1963; Carton and Montgomery 2004; Webb 1989a,b; Teyke 1990; Vischer 1990; Honkanen 1993; Wark and Peichel 2010; Wellenreuther *et al.* 2010; Trokovic *et al.* 2011). This variation has been correlated with behavioral differences (Carton and Montgomery 2004) and with ecological and hydrodynamic features of aquatic habitats (Coombs *et al.* 1988; Webb 1989a,b; Janssen 1996; Wark and Peichel 2010; Yoshizawa *et al.* 2010; Wellenreuther *et al.* 2010). Recently, divergence in neuromast number and morphology has been functionally linked to differences in the ability to feed in the dark between surface- and cave-dwelling tetras (Yoshizawa *et al.* 2010). These data support the hypothesis that divergence of the lateral line sensory system across species with unique habitats, behaviors, and life histories plays a role in adaptation to different environments (Braun and Grande 2008; Wark and Peichel 2010; Greenwood 2010; Yoshizawa *et al.* 2010).

To examine the genetic architecture of lateral line divergence, we used the threespine stickleback (*Gasterosteus aculeatus*), a small teleost fish that has been widely used as a model to investigate the genetic changes that underlie phenotypic evolution (Peichel *et al.* 2001; Colosimo *et al.* 2004, 2005; Cresko *et al.* 2004; Shapiro *et al.* 2004; Kimmel *et al.* 2005; Miller *et al.* 2007; Albert *et al.* 2008; Kitano *et al.* 2009; Chan *et al.* 2010; Greenwood *et al.* 2011). Stickleback populations occupy a range of habitats, and differences in stickleback lateral line sensory morphology are correlated with differences in ecological conditions (Wark and Peichel 2010). For example, in two different lakes, bottom-feeding “benthic” lake populations show consistently higher numbers of neuromasts than open-water “limnetic” populations (Wark and Peichel 2010). Evolution of similar phenotypes in independent populations implies that there is selection on the lateral line system (Endler 1986; Schluter 2000). However, the genetic basis of these differences has not previously been investigated. In the present study, we compared the lateral line morphology of lab-reared sticklebacks from the Paxton Benthic lake population with the Japanese Pacific Ocean marine population, which represents the ancestral state. These populations live in very different ecological conditions and differ in a number of behaviors, including behaviors mediated by the lateral line, such as schooling (Wark *et al.* 2011). We analyzed three aspects of lateral line morphology: groove prominence in the supraorbital line; neuromast number across 12 anatomically distinct lines of superficial neuromasts; and neuromast patterning in the main trunk line. To identify regions of the genome that contribute to variation in these traits, we performed a quantitative trait locus (QTL) analysis on an F2 intercross between Paxton Benthic and Japanese Pacific marine sticklebacks.

## MATERIALS AND METHODS

### Stickleback crosses and care

Japanese Pacific and Paxton Benthic sticklebacks were bred in the laboratory to generate age-matched clutches. All offspring were raised in identical laboratory conditions without parental care. For the population comparison, both populations were raised together in common garden tanks (Wark *et al.* 2011). For genetic mapping, an *in vitro* cross was made between a single, wild-caught Paxton Benthic female stickleback and a single, first-generation lab-raised Japanese Pacific male stickleback to generate an F1 family. Four F1 females were independently crossed to four F1 male siblings to generate four F2 families.

All sticklebacks were housed in 29-gallon aquarium tanks under summer lighting conditions (16 hr light, 8 hr dark) at approximately 15.5°. Tanks were filled with stickleback aquarium water (0.35% saltwater: 3.5g/l Instant Ocean salt, 0.4 ml/l NaHCO3). Water was oxygenated with an air stone and circulated through an external charcoal filter (AquaClear 20 Power Filter; Hagen, Montreal, Canada). Fish were fed live *Artemia* nauplii twice daily. All animal procedures were approved by the Fred Hutchinson Cancer Research Center Institutional Animal Care and Use Committee (protocol 1575).

### Neuromast visualization and analysis

Eight Japanese Pacific sticklebacks, 8 Paxton Benthic sticklebacks, and 236 F2 hybrid sticklebacks were examined for lateral line morphology at approximately one year of age. Average standard lengths in cm ± SEM of the fish were: Japanese Pacific (5.0 ± 0.06), Paxton Benthic (5.0 ± 0.15), and F2s (4.72 ± 0.03). To count neuromasts, fish were stained with the fluorescent vital dye 2-(4-(dimethylamino)styrl)-N-ethylpyridinium iodide (DASPEI; Invitrogen/Molecular Probes, Carlsbad, CA). Live fish were placed in aerated 0.025% DASPEI in 30% tank water and 70% deionized water for 15 min. Fish were then deeply anesthetized in 0.016% MS-222 (tricaine methylsulfonate; Fisher Scientific, Pittsburgh, PA) for approximately 5 min, or until the fish were motionless and breathing very shallowly. Fish were gently submerged in a Petri dish containing 0.005% MS-222 and mounted on a Leica fluorescence dissecting scope with a FITC filter set (Leica Microsystems Inc., Bannockburn, IL). Neuromasts were counted in all 12 lines that compose the stickleback lateral line system (Wark and Peichel 2010). Abbreviations for these lines are as follows: mandibular (MD), ethmoid (ET), supraorbital (SO), infraorbital (IO), oral (OR), preopercular (PO), otic (OT), anterior pit (AP), supratemporal (ST), main trunk line anterior (Ma), main trunk line posterior (Mp), and caudal fin (CF). Only neuromasts on the left side of the body were counted. Following staining and neuromast quantification, fish were returned to 0.016% MS-222 and killed. Fin tissue was extracted and placed in ethanol for subsequent DNA extraction. Bodies were placed in 10% buffered formalin.

To quantify neuromast patterning in the main trunk line, neuromasts in each body segment (myomere) were categorized according to the primary axis of patterning: dorso-ventral (vertical distribution) or anterior-posterior (horizontal distribution). Neuromast distribution could only be determined when sufficient neuromasts were present in a given body segment. In Ma, two neuromasts were required for classification because the dorsal-ventral midline was difficult to determine and neuromast position had to be compared relative to one another. In Mp, segments with single neuromasts could be categorized because the midline of each body segment could easily be observed, regardless of plating. A summary ratio of dorso-ventral patterning was calculated by dividing the number of segments with a vertical neuromast distribution by the number of segments that could be phenotyped.

DASPEI staining was not consistent across the body in all F2 hybrids, due to unequal stain penetration or high background. For each individual, any lines in which neuromasts could not be clearly visualized were excluded from the QTL data set. Furthermore, 32 F2 hybrids had weak or inconsistent staining on the majority of the body. These animals were used to make the linkage map and in the QTL analysis for skeletal traits, but they were excluded from the QTL analysis for neuromast number and pattern.

### Groove morphology

Groove depth in the supraorbital (SO) line was scored on a fluorescent stereomicroscope during DASPEI staining. Grooves were assigned a score based on qualitative observations of depth, ranging from 0 (no grooves detected) to 3.5 (deepest grooves observed).

### Skeletal trait characterization

Lateral plates were visualized by staining all F2 hybrids with alizarin red (Fisher Scientific, Pittsburgh, PA), a calcium stain. Fish were removed from formalin and placed in dH_2_0 overnight. Fish were placed in 0.008% alizarin red in 1% KOH for 24 hr and then de-stained in several washes of dH_2_0. Plates were counted on the left side of the body. In addition to total plate number, the number of plates in the body regions corresponding to the anterior and posterior portions of the main trunk line were recorded. This boundary is defined as the position where the last plate in Ma contacts both the support structure for the second dorsal spine and the pelvic girdle. Animals were also assessed for the presence of a pelvic girdle and pelvic spines. Animals with a complete or partial pelvic girdle and pelvic spines were assigned a score of 1, and animals lacking any pelvic structures were assigned a score of 0.

### Imaging

Fluorescent images of neuromasts were captured using a Retiga camera (QImaging, Surrey, BC, Canada). The contrast of these images was adjusted uniformly using the automated “Levels” function in Adobe Photoshop. Alizarin red-stained animals were photographed on a Nikon SMZ1500 light stereomicroscope equipped with a Nikon Coolpix 4500 digital camera (Nikon, Melville, NY). Schematics of F2 hybrid phenotypes were created by overlaying DASPEI images and alizarin red images in Adobe Illustrator.

### Statistics

Statistical analyses were performed in SPSS 13.0 software (SPSS, Chicago, IL). Japanese Pacific and Paxton Benthic neuromast numbers were compared using multivariate analysis of variance (MANOVA). Overall differences among groups were tested with the Wilks lambda multivariate test. Epistatic interactions between linkage groups (LG) 4 and 21 were assessed by ANOVA.

### Quantitative trait locus analysis

Genomic DNA was isolated from fin clips using phenol-chloroform extraction, followed by ethanol precipitation and resuspension in 50 μl TE (10 mM Tris, 1 mM EDTA). Both grandparents, 7 F1 parents, and 236 F2 hybrids were genotyped using 1536 genome-wide single nucleotide polymorphism (SNP) markers on a custom-built stickleback Golden Gate SNP array (Illumina, San Diego, CA; Jones *et al.* 2012a). SNP genotypes were analyzed using GenomeStudio software (Illumina). There were 245 SNPs with fixed differences between the Paxton Benthic and Japanese Pacific grandparents (Table S1); these were combined with five microsatellite markers on LG 21 (Table S2) to create a linkage map with JoinMap 3.0 (Van Ooijen and Voorrips 2001). The linkage map consisted of 22 linkage groups, including 2 linkage groups containing markers from chromosome 14 (labeled 14a and 14b). Three markers did not associate with any linkage groups, leaving 247 markers in the final map. Two F2 individuals had poor genotyping data and were excluded from the study, leaving 234 F2 hybrids in the QTL mapping analysis. All genotype and phenotype data for these 234 F2s are provided in File S1.

Interval mapping was performed using MapQTL 4.0 (Van Ooijen *et al.* 2002). Because we focused on identifying QTL segregating between the Paxton Benthic and Japanese Pacific populations, only markers with fixed differences between the grandparents were used for QTL mapping (*i.e.*, all F1s were heterozygous), and data for all four F2 families were therefore combined in the analysis. Genome-wide likelihood of odds (LOD) significance thresholds were established for each trait using permutation testing (*α* = 0.05, 1000 permutations). When permutation testing could not be used (pelvis, Ma pattern, and Mp pattern for plated segments only), we employed a conservative genome-wide LOD significance threshold of 4.2 (*α* = 0.05), based on simulations for an F2 population (Van Ooijen 1999). Only QTL that met genome-wide significance thresholds at a nearby marker are reported.

## RESULTS AND DISCUSSION

### Japanese Pacific and Paxton Benthic sticklebacks differ in lateral line morphology

**Figure 1:**
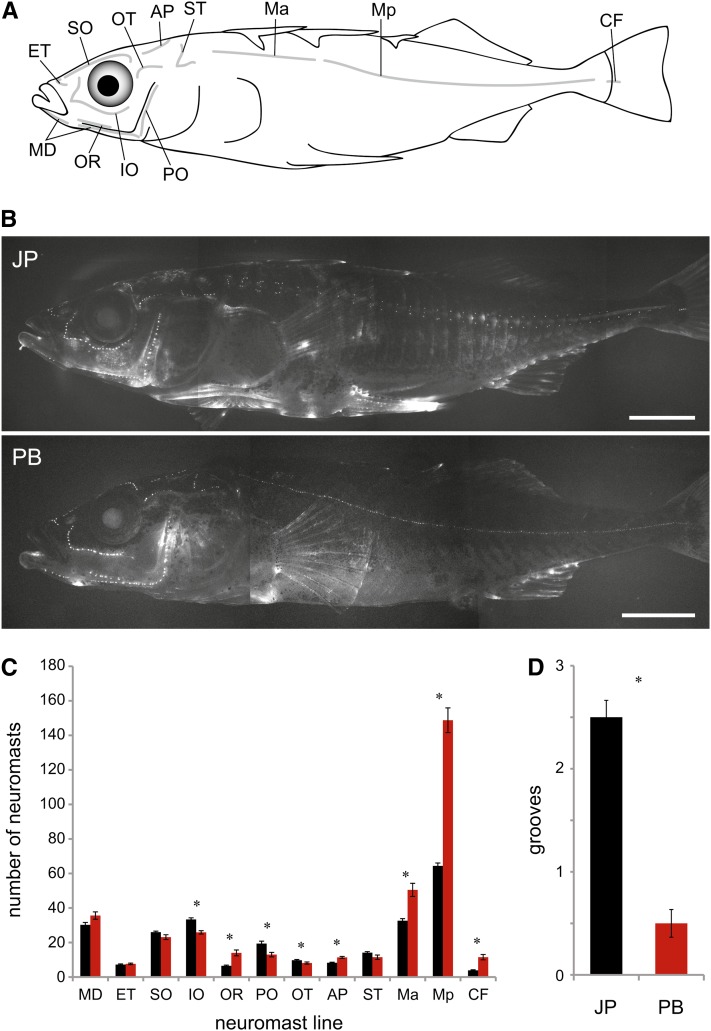
Laboratory-raised Japanese Pacific and Paxton Benthic sticklebacks differ in neuromast number and groove morphology. (A) Schematic diagram of the 12 lines of neuromasts in the threespine stickleback lateral line, reproduced from Wark and Peichel (2010). See text for neuromast line abbreviations. (B) Images of DASPEI-stained Japanese Pacific (JP) and Paxton Benthic (PB) sticklebacks. Neuromasts are observed as punctate spots. The outlines of the bony lateral plates found in the region of the Ma and Mp lines can be seen in the Japanese Pacific fish. Scale bars = 0.5 cm. (C) Neuromast number in each line in Japanese Pacific (black) and Paxton Benthic (red) sticklebacks. (D) Groove depth in Japanese Pacific (black) and Paxton Benthic (red) populations. *N* = 8 for each population. Asterisks indicate significant differences between groups (*P* < 0.05). Error bars = standard error.

We compared the lateral line morphology of eight Japanese Pacific marine and eight Paxton Benthic lake sticklebacks raised in identical laboratory conditions. We assessed three aspects of lateral line morphology: supraorbital (SO) groove morphology, neuromast number in each of the 12 superficial neuromast lines found in threespine sticklebacks (Figure 1A), and neuromast patterning in the anterior and posterior portions of the main trunk line (Ma and Mp).

We previously showed that neuromasts of the SO line sit in a groove that is depressed below the surrounding skin in wild-caught Japanese Pacific sticklebacks, whereas neuromasts in the SO line in wild-caught Paxton Benthic sticklebacks sit flush with the skin (Wark and Peichel 2010). Here, we have quantified that difference in laboratory-reared fish using a groove rating score ranging from 0 (no grooves detected) to 3.5 (deepest grooves observed). Using this index, Japanese Pacific sticklebacks have significantly deeper grooves than Paxton Benthic sticklebacks (*F*_1,14_ = 89.60; *P* < 0.001; Figure 1D). Although the function of these grooves in marine sticklebacks has not been investigated, it has been suggested that they resemble partially formed canals that could act as a sensory filter affecting perception and behavior (Wark and Peichel 2010), as has been observed for the fleshy ridges surrounding cephalic neuromasts in killifish (Schwarz *et al.* 2011).

Japanese Pacific and Paxton Benthic sticklebacks have different numbers of neuromasts across the body (*F*_1,14_ = 38.36, *P* < 0.01; Figure 1, B and C). Japanese Pacific sticklebacks have more neuromasts in several lines that are located on the head: IO (*P* < 0.001), OT (*P* = 0.04) and PO (*P* = 0.005). Paxton Benthics have more neuromasts in lines that are predominantly located more caudally: OR (*P* < 0.001), Ma (*P* < 0.001), Mp (*P* < 0.001), CF (*P* < 0.001) and AP (*P* < 0.001). Neuromast number did not differ significantly between the two populations in four lines: ET (*P* = 0.53), MD (*P* = 0.051), SO (*P* = 0.079), and ST (*P* = 0.086).

**Figure 2:**
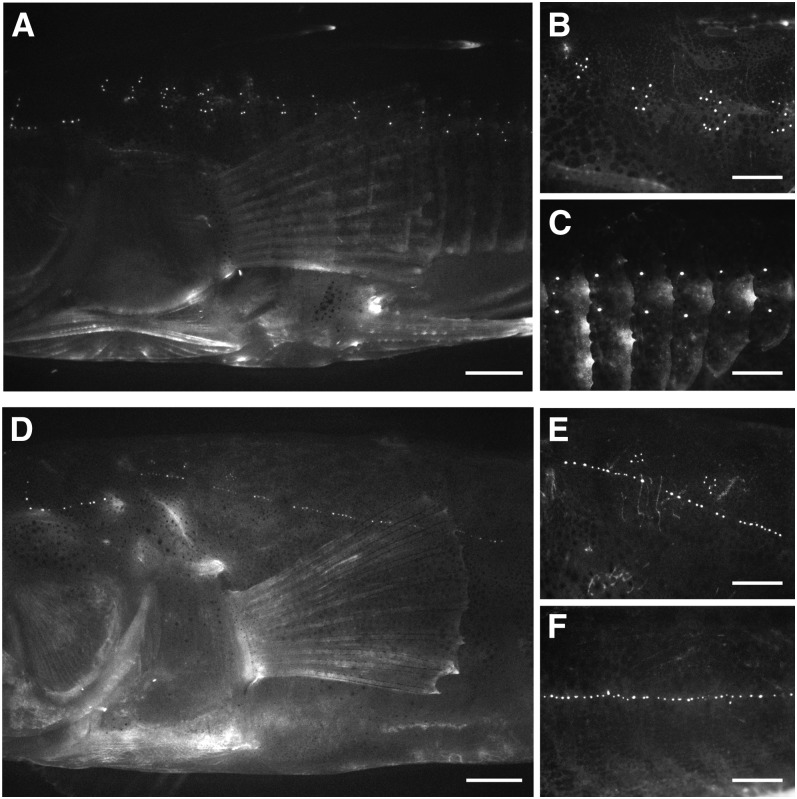
Anterior and posterior main trunk line patterning differs between Japanese Pacific and Paxton Benthic sticklebacks. Lateral views of main trunk line neuromast patterning in DASPEI-stained Japanese Pacific (A–C) and Paxton Benthic (D–F) sticklebacks. Magnified views (B, E) of the Ma line. Magnified views (C, F) of the Mp line. Scale bars = 0.2 cm (A, D); 0.1 cm (B, C, E, F). Bony lateral plates on the Japanese Pacific fish can be seen most clearly in panel C.

These marine and benthic lake populations also differ in patterning of the neuromasts in the main trunk line. Here, differences in neuromast patterning are associated with differences in the presence of the bony lateral plates, which occupy both sides of the body in threespine sticklebacks (Figures 1B and 2). Japanese Pacific sticklebacks are “completely” plated, with plates extending across the body segments encompassed by both the Ma and Mp lines, ending in a bony keel at the caudal peduncle. Paxton Benthic sticklebacks are “low” plated, meaning that they only have plates in the region of the Ma line, ending at or before the second dorsal spine (Colosimo *et al.* 2004). In plated body segments of both complete and low-plated sticklebacks, neuromasts are situated on the plates (Figure 2C; Wark and Peichel 2010).

In the Ma line, Japanese Pacific sticklebacks exhibit a vertical distribution of neuromasts on each body segment, with individual neuromasts primarily located dorsally and ventrally from the midpoint of each lateral plate (Figure 2, A and B). In contrast, Paxton Benthic sticklebacks have few or no plates in this region and exhibit a predominantly horizontal distribution of neuromasts in Ma, with a small number of neuromasts located dorsal to a nearly continuous horizontal row of neuromasts (Figure 2, D and E). In the Mp line, Japanese Pacific sticklebacks exhibit a dorso-ventral distribution of neuromasts relative to the midpoint of each lateral plate (Figure 2, A and C). Here, Mp neuromasts are frequently organized as pairs or triplets on each body segment rather than the larger clusters observed in Ma. Paxton Benthic sticklebacks are unplated in the Mp region, and the neuromasts are not arranged in a dorso-ventral pattern. Rather, the Mp neuromasts are arranged in an anterior to posterior distribution that often appears continuous across body segments (Figure 2, D and F). An association between the presence of plates and the dorso-ventral distribution of neuromasts has been observed across multiple stickleback populations (Wark and Peichel 2010), but the genetic and developmental mechanisms that underlie this association were unknown.

### Genetic architecture of divergence in lateral line morphology

Differences in groove morphology, neuromast number, and neuromast patterning were all observed in lab-raised individuals, suggesting that the differences between these populations have a heritable component. To identify genetic loci contributing to differences in the morphology of the lateral line, we performed a QTL analysis on 236 Paxton Benthic × Japanese Pacific F2 hybrid sticklebacks. Analysis of lateral line and skeletal phenotypes segregating in F2 offspring identified 23 QTL affecting groove morphology, neuromast number, neuromast pattern, the presence of lateral plates, and the presence of pelvic structures. The chromosome positions and phenotypic effect sizes of all QTL are summarized in Table 1.

**Table 1:** Significant QTL for lateral line and skeletal traits

					Phenotypic Means			
Trait	LG	LOD	Marker	Position (cM)	JP/JP	JP/PB	PB/PB	PVE
Neuromast number								
MD	5	3.6	chrV:8214190, chrV:8211082	32.7	30.70 ± 4.47	28.08 ± 4.71	31.27 ± 5.39	8.7
SO	8	4.0	chrVIII:12472630	19.2	23.34 ± 5.87	24.40 ± 4.93	27.50 ± 3.38	8.9
OR	11	3.5	chrXI:5472842–chrXI:5845760*^a^*	0.0	8.13 ± 3.10	7.45 ± 1.86	6.28 ± 1.93	7.9
ST	4	4.9	chrIV:27614532	60.0	11.55 ± 3.26	10.43 ± 3.41	8.12 ± 3.30	11.8
	5	4.2	chrV:8327818	33.4	10.71 ± 3.39	9.07 ± 2.87	11.60 ± 4.14	10.0
	12	4.9	chrXII:14346080, chrXII:14353450	29.5	9.04 ± 3.73	9.90 ± 3.15	12.58 ± 3.30	11.7
Ma	11	3.6	chrXI:9039275	5.1	24.11 ± 9.03	19.48 ± 7.78	17.58 ± 6.77	8.7
	13	5.9	chrXIII:17896505	47.1	17.72 ± 7.85	19.02 ± 5.68	25.90 ± 11.10	13.9
Mp	21	15.2	chrXXI:7904439	9.2	29.59 ± 7.91	35.24 ± 11.7	55.85 ± 19.04	36.0
CF	11	5.3	chrXI:9039275	5.1	4.25 ± 2.34	2.60 ± 1.55	2.63 ± 1.54	14.1
Total (all lines)	5	3.9	chrV:10649179	52.9	230.0 ± 50.3	212.9 ± 40.8	251.9 ± 14.3	10.9
Neuromast pattern								
Ma	21	20.0	chrXXI:4004587, chrXXI:4500405	7.1–7.3	0.98 ± 0.07	0.97 ± 0.07	0.70 ± 0.27	39.6
Mp	4	41.3	chrIV:12817401	50.5	0.90 ± 0.25	0.62 ± 0.33	0.0 ± 0.0	64.8
	21	5.4	chrXXI:4500405	7.3	0.66 ± 0.41	0.57 ± 0.42	0.24 ± 0.34	12.8
Mp (plates only)	21	8.3	chrXXI:4500405	7.3	0.93 ± 0.14	0.90 ± 0.18	0.62 ± 0.29	25.3
Skeletal traits								
Grooves	21	4.9	chrXXI:5716516, chrXXI:5793103, chrXXI:7002178	8.8	1.06 ± 0.73	0.83 ± 0.78	0.43 ± 0.56	9.1
Ma plates	4	39.0	chrIV:12817401	50.5	6.92 ± 0.38	6.68 ± 0.59	5.21 ± 0.92	53.6
	21	8.9	chrXXI:4500405	7.3	6.63 ± 0.72	6.50 ± 0.74	5.62 ± 1.29	16.0
Mp plates	4	73.4	chrIV:12817401	50.5	24.86 ± 3.86	18.12 ± 6.83	0.46 ± 0.59	79.5
	21	6.2	chrXXI:4500405	7.3	17.79 ± 10.24	16.13 ± 10.51	7.47 ± 9.29	12.5
Total plates	4	74.5	chrIV:12817401	50.5	31.78 ± 4.14	24.79 ± 7.14	5.67 ± 1.21	80.0
	21	6.6	chrXXI:4500405	7.3	24.38 ± 10.83	22.60 ± 11.12	13.00 ± 10.26	13.3
Pelvis	7	42.0	chrVII:27757015	95.8	0.97 ± 0.18	0.94 ± 0.24	0.21 ± 0.41	65.6

For each QTL, the linkage group (LG), likelihood of odds (LOD), name of marker(s) with the highest LOD score, genetic map position in centiMorgans (cM), phenotypic means for each genotype class (mean ± SD), and percentage variance explained (PVE) are provided. See text for neuromast line abbreviations.

a LG11 QTL region encompasses six markers (chrXI:5472842, chrXI:5652180, chrXI:5653380, chrXI:5708414, chrXI:5845597, and chrXI:5845760).

#### Modularity:

**Figure 3:**
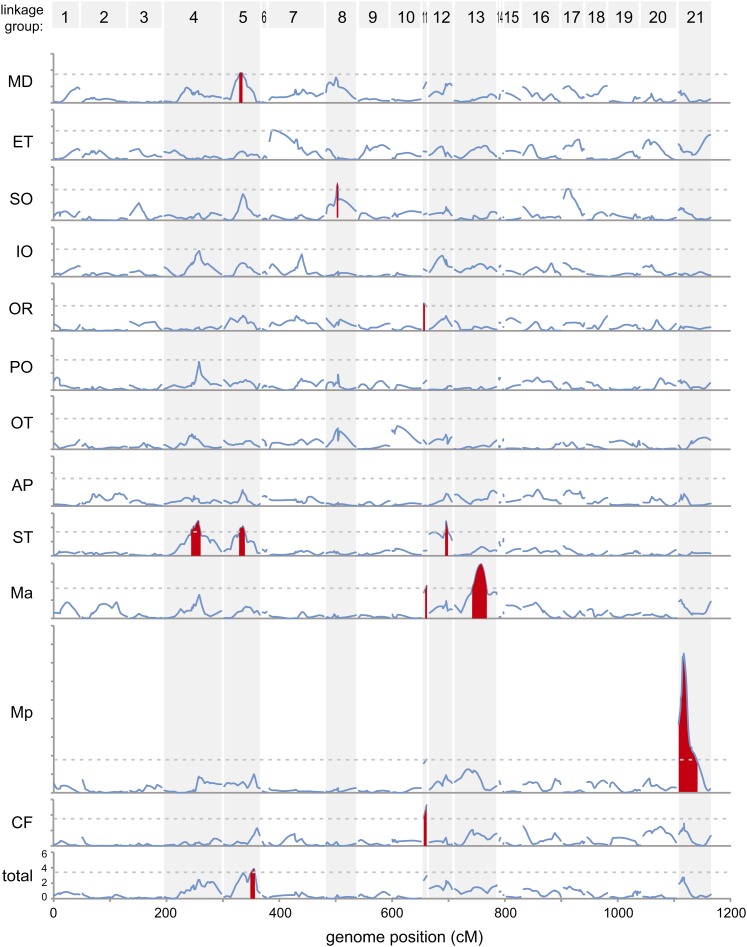
Significant QTL for neuromast number. LOD scores are shown in sequential graphs for each neuromast line (scale range: LOD = 0–18 for Mp, LOD = 0–6 for all others) relative to genomic position in cM. Linkage groups are shown in ascending order from left to right, numbered at the top. Dashed lines indicate genome-wide significance thresholds for each neuromast line. Red peaks indicate significant QTL. Linkage groups with significant QTL for one or more neuromast lines are highlighted with vertical gray bars.

A striking aspect of the results is that many of the QTL affect lateral line morphology in specific body regions, rather than throughout the lateral line system. For example, QTL in seven genomic regions influence neuromast number. Each of these QTL acts in separable subsets of the overall lateral line system, rather than causing a global increase or decrease in neuromast number throughout the body (Table 1; Figure 3). Particularly striking is the fact that the number of neuromasts in the Ma line is linked to completely different QTL than the number of neuromasts in the Mp line (Table 1; Figure 3). These results argue against a genetic mechanism that causes global proliferation or reduction of neuromasts. Rather, these QTL mapping results provide evidence for a modular genetic architecture that could facilitate the evolution of regional specializations of the lateral line sensory system in sticklebacks that have adapted to different environments (Schlosser and Wagner 2004). Developmental studies of the lateral line system in other species further support the idea that each line is a distinct developmental module (Modrell *et al.* 2011). It is too early to know whether such a modular genetic architecture is a common feature of sensory systems, as very few QTL studies have been conducted on sensory systems (Mackay and Lyman 2005; Carleton *et al.* 2010).

#### Size and direction of effects:

QTL detected on LG 4 and LG 21 had major effects on neuromast phenotypes, ranging from 25 to 65% of the variance explained for neuromast number or patterning in particular body regions. Most other chromosome regions had smaller phenotypic effects, ranging from 8 to 15% of variance explained. These results are consistent with both theoretical work and other QTL mapping studies, showing that many evolutionary differences are controlled by QTL with a range of effect sizes, including a small number of loci with substantial phenotypic effects (Orr 1999, 2005; Colosimo *et al.* 2004; Shapiro *et al.* 2004; Albert *et al.* 2008).

Most of the QTL we detected appear to act semi-additively: F2 fish that are heterozygotes have phenotypes that are intermediate to those seen in fish homozygous for either the Japanese Pacific or Paxton Benthic alleles (Table 1). However, there is evidence of underdominance at QTL on LG 5 (MD, ST, and total neuromast number) because heterozygotes have fewer neuromasts than either class of homozygotes, and there is evidence for dominance at a QTL on LG 11 (CF line) as heterozygotes have the same phenotypic mean as Paxton Benthic homozygotes (Table 1).

The QTL with the biggest phenotypic effects also had directions of effects that were consistent with the overall phenotypic difference between Japanese Pacific and Paxton Benthic sticklebacks. For example, substituting Paxton Benthic alleles at the LG 21 QTL caused a progressive increase in the average number of Mp neuromasts in the F2 progeny (Table 1), a result consistent with the higher number of neuromasts also seen in the parental Paxton Benthic population (Figure 1C). QTL with smaller phenotypic effects sometimes had directions of effects that did not match the known phenotypic difference between Japanese Pacific and Paxton Benthic sticklebacks; for example, F2 individuals homozygous for Japanese Pacific alleles on LG 11 had more neuromasts in both the Ma, OR, and CF lines (Table 1), whereas Paxton Benthic sticklebacks had more neuromasts in these lines (Figure 1C).

It is important to note that our ability to obtain a comprehensive view of the genetic architecture of variation in the lateral line was limited by the relatively small size of our cross and the resolution of our map (Beavis 1998; Otto and Jones 2000; Noor *et al.* 2001). Nevertheless, the existence of many small QTL with mixed directional effects is consistent with previous QTL mapping studies in natural populations (Rieseberg *et al.* 2002). These data suggest that there is within-population variation for genes that affect the lateral line, consistent with the previously reported variation in neuromast number within stickleback populations (Wark and Peichel 2010). It is also likely that there are some environmental effects on neuromast number. For example, we did not detect any QTL for the number of neuromasts in the IO, PO, and OT lines, although Japanese Pacific fish had significantly more neuromasts in these lines than the Paxton Benthic fish (Figure 1C). However, in our previous study, wild-caught Paxton Benthic fish had more neuromasts than Japanese Pacific fish in these lines (Wark and Peichel 2010), suggesting that genetic variation within the populations and/or environmental factors contribute to phenotypic variation in neuromast number within stickleback populations. Importantly, however, we did detect QTL with large and consistent effects on neuromast phenotypes, particularly on LG 4 and LG 21.

#### Constructive and regressive traits:

Most previous studies reporting QTL of large effects in sticklebacks have focused on differences where freshwater fish have lost structures or cells found in marine ancestors, including loss of armor plates, loss of pelvic structures, or reduction of pigmentation (Colosimo *et al.* 2004, 2005; Cresko *et al.* 2004; Shapiro *et al.* 2004; Miller *et al.* 2007; Chan *et al.* 2010). By contrast, the dramatic expansion of neuromast number in the Mp line of Paxton Benthic fish is an excellent example of a “constructive” rather than a “regressive” trait, one based on an increase or gain of structures, rather than reduction or loss. Interestingly, the LG 21 QTL for Mp neuromast number has the largest effect size of any of the QTL influencing neuromast number, and increasing substitution of Paxton Benthic alleles at this QTL leads to an increased rather than decreased number of neuromasts (Table 1). This LG 21 QTL, explaining nearly a third of the variance in neuromast number, thus represents a major locus influencing constructive evolutionary change in sticklebacks. Increased numbers of Mp neuromasts per body segment have been observed in two independent stickleback populations adapted to benthic environments (Wark and Peichel 2010), suggesting that there is likely to be selection for this phenotype in benthic sticklebacks (Endler 1986; Schluter 2000). Future experiments are aimed at identifying both the selective advantage of additional Mp neuromasts in the benthic habitat, as well as the actual gene or genes that underlies this constructive evolutionary QTL.

#### Epistatic interactions between LG 4 and LG 21:

**Figure 4:**
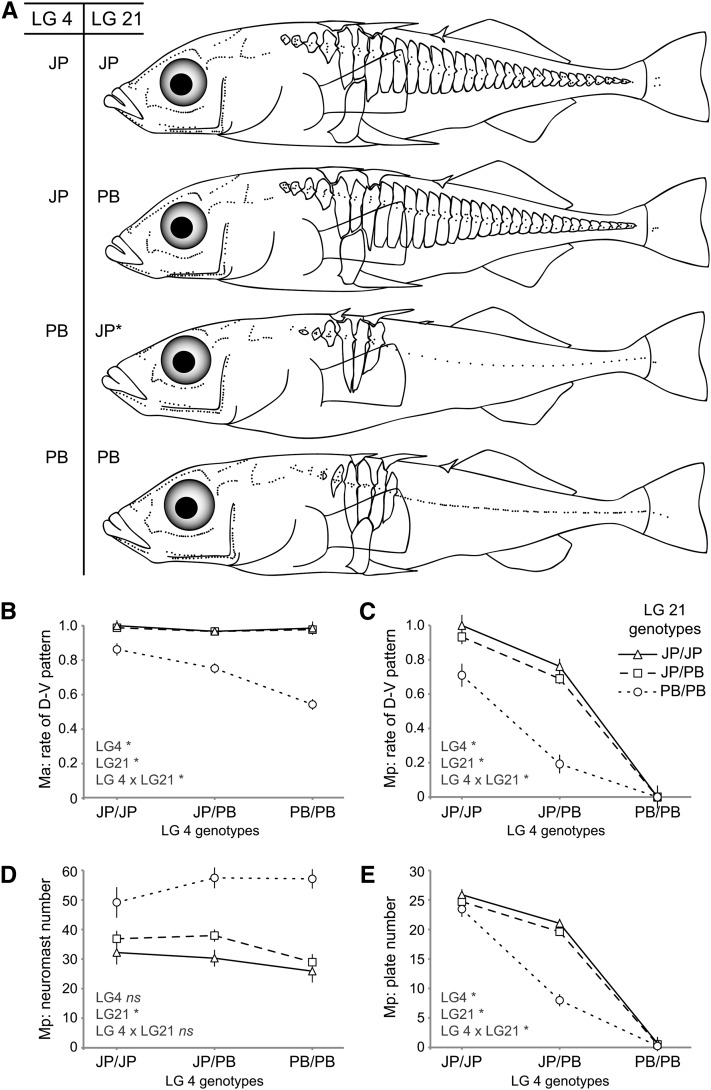
Neuromast patterning in the main trunk line shows significant epistatic interactions between LG 4 and LG 21. (A) Skeletal morphology and neuromast distribution of individual F2s of various genotypes. Each dot represents a single neuromast. Fin size and shape are approximate. The LG 4 genotypes (at marker chrIV:12817401) and LG 21 genotypes (at marker chrXXI:4500405) are indicated at the left. PB, Paxton Benthic homozygote, JP, Japanese Pacific homozygote; JP*, JP/PB heterozygote, where JP allele acts dominantly. Note that the third F2 lacks a pelvic girdle; loss of the pelvic girdle maps to LG 7 in this study (Table 1), as in previous QTL mapping experiments using the same populations (Shapiro *et al.* 2004). Graphs summarize the rate of dorso-ventral distribution for neuromasts in Ma (B) and Mp (C), number of neuromasts in Mp (D), and lateral plate number in Mp (E) for F2s of various genotypic classes. The LG 4 genotypes (at marker chrIV:12817401) are shown on the x-axis. The LG 21 genotypes (at marker chrXXI:4500405 in panels B, C, and E; at marker chrXXI:7904439 in panel D) are indicated as follows: triangles with solid lines = JP/JP; squares with dashed lines = JP/PB; circle with dotted lines = PB/PB; and error bars = SEM. Significant effects of LG 4, LG 21, or their interaction are indicated with an asterisk; non-significant effects are indicated as *ns*; see text for statistics.

The two QTL of largest effect in our data display epistatic interactions that influence neuromast pattern along the anterior and posterior segments of the main trunk lateral line. Fish with Japanese Pacific alleles at the QTL on LG 4 and LG 21 have typical marine-like patterns (Figure 4A). Substitution of Paxton Benthic alleles at the LG 21 locus has a small but significant effect on the dorso-ventral patterning of neuromasts in the Ma line, with a much larger effect in fish that also carry freshwater alleles at the LG 4 locus [Figure 4B; *F*_interaction_ (*df* = 4, error = 174) = 8.817, *P* < 0.001]. Epistatic interactions between these loci have even more dramatic effects in the Mp line. Fish that are homozygous for Paxton Benthic alleles at LG 4 fail to exhibit dorso-ventral distribution of neuromasts in Mp regardless of their genotype at LG 21 [Figure 4C; *F*_interaction_ (*df* = 4, error = 173) = 8.904, *P* < 0.001]. Although there is not a significant interaction between LG 4 and LG 21 for Mp neuromast number [Figure 4D; *F*_interaction_ (*df* = 4, error = 148) = 1.448, *P* = 0.22], the effects of the LG 21 QTL on Mp neuromast number can most readily be observed when fish have two Paxton Benthic alleles at the LG 4 QTL: fish with at least one Japanese Pacific allele on LG 21 have a single neuromast per body segment, whereas fish with two Paxton Benthic alleles on LG 21 have multiple neuromasts per body segment (Figure 4A).

### Genetic association between neuromast pattern and lateral plates

**Figure 5:**
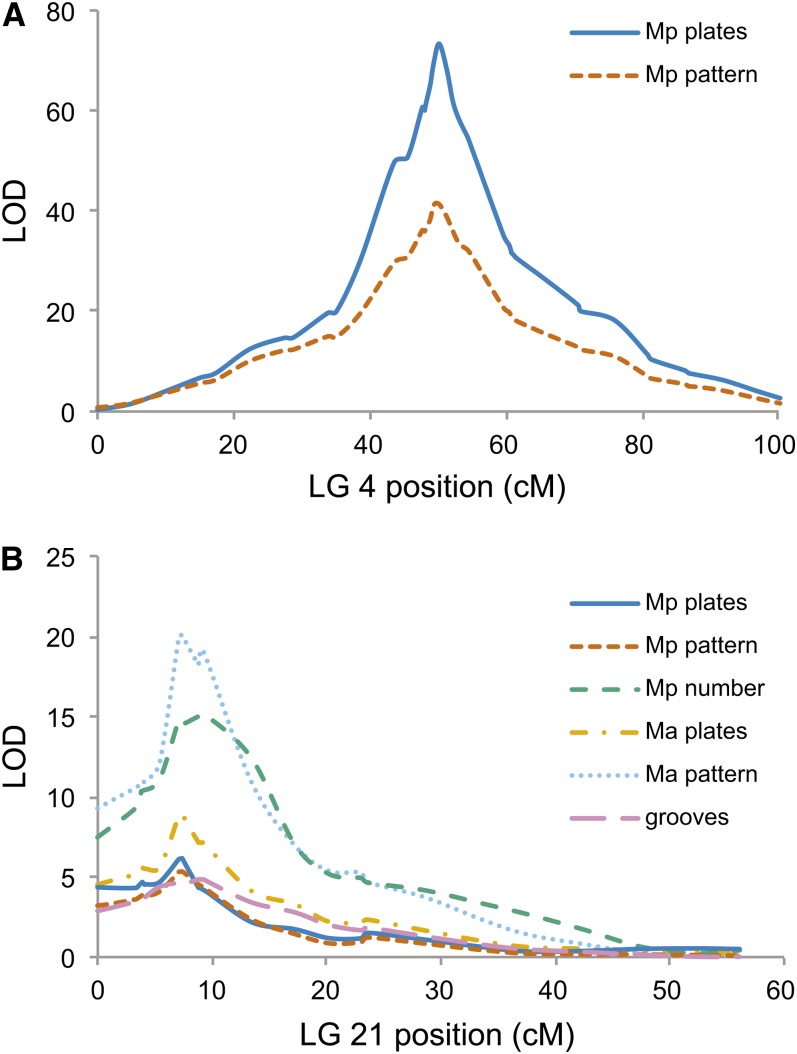
Significant QTL for grooves, neuromast number, neuromast pattern, and lateral plate number map to LG 4 and LG 21. For each trait, LOD scores are plotted relative to genetic map position (cM) on LG 4 (A) and LG 21 (B). The names of the genetic markers and their genetic map positions are provided in Table S1 and Table S2.

Previous studies have shown that LG 4 and LG 21 also contain major QTL controlling lateral plate development, and these QTL also show epistatic interactions for plate number similar to those seen here for neuromast pattern (Colosimo *et al.* 2004). We also mapped lateral plate number in the current cross and found that the major QTL for both lateral plate number and neuromast patterning in the Mp line map to the same locations on LG 4 and LG 21, with similar epistatic effects (Figure 4E) and LOD profiles (Figure 5). The *Ectodysplasin* (*Eda*) gene is located at the peak of the LG 4 QTL, and it was previously shown to be responsible for the presence of plates in the region of the Mp line (Colosimo *et al.* 2005). Given that the dorso-ventral distribution of neuromasts in the Mp line also maps to this region, it is likely that there is a relationship between variation in the *Eda* gene and variation in neuromast pattern. It is possible that *Eda* has pleiotropic effects both on the presence of lateral plates and on neuromast pattern. Alternatively, it is possible that there is a developmental relationship between the presence of lateral plates and the patterning of neuromasts in the Mp line; interactions between bony dermal structures and neuromast patterning during development have been observed in other fish (Wada *et al.* 2010).

To further explore the relationship between neuromast patterning and lateral plates, we examined individual body segments of 159 F2s for the presence of lateral plates and the dorso-ventral distribution of neuromasts in the Mp line. Of the 1713 body segments analyzed that did not have a lateral plate, only 4 (0.23%) had a dorso-ventral distribution of neuromasts, whereas of the 1915 body segments analyzed that did have a lateral plate, 92.2% had a dorso-ventral distribution of neuromasts (Table 2). These data strongly suggest that the presence of a plate is necessary but not sufficient for the dorso-ventral patterning of neuromasts in the Mp line. Furthermore, when we consider only plated body segments, F2s that are homozygous for Paxton Benthic alleles on LG 21 have a reduced rate of dorso-ventral patterning (Table 1), suggesting that additional genetic and developmental mechanisms contribute to neuromast patterning. Additional experiments are currently under way to further disentangle the genetic and developmental mechanisms that underlie the relationship between lateral plates and neuromast patterning.

**Table 2:** Relationship between lateral plate presence and neuromast pattern in the Mp line

	Number of Segments with Neuromast Pattern		
	Dorso-Ventral	No Dorso-Ventral	Total
Plated segments	1765	150	1915
Unplated segments	4	1709	1713
Total segments	1769	1859	3638

Each body segment in the Mp line of 159 F2s with robust DASPEI staining was analyzed for the presence or absence of lateral plates, as well as the dorso-ventral distribution of neuromasts. Only body segments with at least one neuromast were counted. Within a body segment, there was a significant association between the presence of a plate and the presence of a dorso-ventral distribution of neuromasts (χ^2^_(1)_ = 3058.7; *P* < 0.001). Plated segments with no dorso-ventral distribution of neuromasts and unplated segments with a dorso-ventral distribution of neuromasts appeared to be randomly distributed along the anterior-posterior axis.

### Multiple QTL map to an inversion on LG 21

Multiple lateral line-related traits, including grooves, the number of lateral plates, neuromast pattern, and neuromast number, map to overlapping regions on LG 21 (Figure 5B). At the level of resolution provided by our cross, it is difficult to determine whether such clustering is due to pleiotropy or tight linkage. The 2-LOD confidence intervals for these QTL on LG 21 overlap with a chromosomal inversion between Japanese Pacific and Paxton Benthic sticklebacks (Jones *et al.* 2012b). This inversion spans from 5.8 to 7.5 Mb; in our cross, markers at 5.71 Mb, 5.79 Mb, and 7.00 Mb do not recombine with each other (8.8 cM on the genetic map), although they do recombine with flanking markers at 4.50 Mb (7.3 cM) and 7.90 Mb (9.2 cM; Table S1 and Table S2). Although loss of recombination within the inversion makes further genetic mapping difficult in the current cross, the fact that multiple traits map to the region of this inversion supports the theoretical prediction that inversions can facilitate linkage of alleles that underlie multiple traits important for adaptation to a new environment (Kirkpatrick and Barton 2006; Jones *et al.* 2012b). As differences in neuromast number are also seen among freshwater populations (Wark and Peichel 2010), some of which share the same inversion orientation on LG 21, additional crosses with freshwater fish should make it possible to further resolve the region or regions controlling multiple phenotypes on LG 21.

Despite the fact that this is still a large QTL region, the rich resources of zebrafish developmental biology suggest a number of promising candidate genes in the LG 21 region for both skeletal and neuromast phenotypes. For example, the *eya1* gene found in this non-recombining region has documented effects on hair cell development in zebrafish (Whitfield *et al.* 1996; Sahly *et al.* 1999; Kozlowski *et al.* 2005). Functional experiments are currently under way to assess the effects of candidate genes in this region of LG 21 on lateral line morphology. The identification of the genes that underlie differences in both neuromast number and pattern will ultimately allow us to dissect the effect of specific genetic changes on lateral line structures, the evolution of constructive traits in freshwater sticklebacks, and the contribution of sensory modifications to behavioral evolution and adaptation in natural environments.

## Supplementary Material

Supporting Information
