# Supplementary material for: Genetic Architecture of Variation in the Lateral Line Sensory System of Threespine Sticklebacks
Source: G3 (Bethesda). 2012 Sep 1;2(9):1047–56. doi: 10.1534/g3.112.003079 (PMC3429919; doi:10.1534/g3.112.003079)
Supplement: Supporting Information [file supp_2_9_1047__index.html]

Supporting Information 

# Genetic Architecture of Variation in the Lateral Line Sensory System of Threespine Sticklebacks

## Supporting Information for Wark *et al.*, 2012

**Files in this Data Supplement:**

- Supporting Information - File S1 and Tables S1 and S2 (PDF, 117 KB)
- Table S1 - SNP markers used for QTL mapping (PDF, 90 KB)
- Table S2 - Microsatellite markers used for QTL mapping (PDF, 48 KB)
- File S1 - Supporting Data (.xls, 861 KB)
