# Supplementary material for: Genetic Architecture of Variation in the Lateral Line Sensory System of Threespine Sticklebacks
Source: G3 (Bethesda). 2012 Sep 1;2(9):1047–56. doi: 10.1534/g3.112.003079 (PMC3429919; doi:10.1534/g3.112.003079)
Supplement: Supporting Information [file supp_2.9.1047_003079SI.pdf]

File S1

Supporting Data

File S1 is avaialble for download at http://www.g3journal.org/lookup/suppl/doi:10.1534/g3.112.003079/-/DC1 as an Excel file.

Table S1 SNP markers used for QTL mapping

Linkage group (LG)	Map position (cM)	Marker name (chromosome:position)	NCBI assay ID (ss#)
1	0	chrI:3310077	244222768
1	10.81	chrI:7820850	244222772
1	11.03	chrI:7955458	120258413
1	11.03	chrI:7955618	252841118
1	12.53	chrI:11963492	120258415
1	12.53	chrI:12038660	120258416
1	37.52	chrI:25201193	252841111
1	46.02	chrI:21511782	244223005
1	46.02	chrI:21951727	252841103
2	0	chrUn:23753128	244222989
2	0.46	chrII:418094	244222780
2	5.3	chrII:2829800	252841149
2	6.49	chrII:3131035	252841121
2	12.12	chrII:4157699	252841112
2	16.64	chrII:5590307	252841076
2	17.79	chrII:5935944	252841148
2	20.58	chrII:7203830	252841170
2	24.23	chrII:8979491	252841135
2	29.97	chrII:13353603	244222783
2	52.6	chrII:19985741	244222785
2	60.8	chrII:20714675	252841046
2	67.68	chrII:21122542	252841085
2	78.99	chrII:22443700	244222787
3	0	chrIII:1651721	252841079
3	16.07	chrIII:10376395	244222790
3	22.43	chrIII:12316694	252841106
3	25.24	chrIII:13520975	252841102
3	30.25	chrIII:14048561	252841058
3	31.55	chrIII:14456990	252841063
3	44.24	chrIII:15783657	252841104
3	48.21	chrIII:16224572	120258430
3	48.26	chrIII:16251071	120258431
3	56.19	chrUn:33873966	252841086
4	0	chrIV:1020296	252841071
4	17.48	chrIV:2858360	252841155
4	28.98	chrIV:3764773	252841050

4	35.29	chrIV:4599765	244222800
4	38.83	chrIV:5867068	252841134
4	45.84	chrIV:8579158	244223069
4	48	chrIV:10812344	244223042
4	48.23	chrIV:10960835	120258434
4	49.09	chrIV:11367975	120258435
4	49.71	chrIV:12005556	252841179
4	50.55	chrIV:12817401	244223048
4	51.58	chrIV:13850026	244223049
4	52.54	chrIV:14325385	252841061
4	53.44	chrIV:15052901	244222804
4	54.74	chrIV:15530121	244222805
4	55.2	chrIV:15737291	244222807
4	60.01	chrIV:27614532	120258442
4	60.81	chrIV:23970813	252841168
4	61.18	chrIV:20957877	252841152
4	61.22	chrIV:21605258	252841082
4	61.84	chrIV:19812956	244223055
4	65.97	chrIV:29763654	120258443
4	71.37	chrIV:30568387	252841083
4	81.21	chrIV:31583885	252841078
4	81.94	chrIV:31611147	252841084
4	87.55	chrIV:32092919	252841132
4	100.95	chrUn:27402745	252841068
5	0	chrUn:11621796	244222985
5	0	chrUn:11980918	252841136
5	11.26	chrV:7791830	252841093
5	23.71	chrV:2528528	244222814
5	32.74	chrV:8211082	252841163
5	32.74	chrV:8214190	252841172
5	33.4	chrV:8327818	244222816
5	41.29	chrV:9768052	252841108
5	52.87	chrV:10649179	252841089
5	58.72	chrV:11316476	252841077
5	63.19	chrUn:25691760	252841096
6	0	chrVI:11873663	120258454
6	3.78	chrVI:13220597	252841044
6	4.88	chrVI:13514193	252841099
6	5.98	chrVI:13682067	252841110

6	7.08	chrVI:14131973	252841117
7	0	chrUn:28671327	244222995
7	0	chrUn:29087782	244222996
7	0	chrVII:537136	252841113
7	0.22	chrVII:286225	252841088
7	4.65	chrVII:835236	252841091
7	44.23	chrVII:5552972	252841066
7	44.47	chrVII:5936068	120258457
7	46.25	chrUn:7866784	252841054
7	47.81	chrVII:13205977	252841062
7	47.81	chrVII:13452516	244222836
7	47.81	chrVII:13525838	244222837
7	49.03	chrVII:15237354	252841075
7	51.37	chrVII:17992851	252841176
7	51.37	chrVII:17994452	252841175
7	51.37	chrVII:17997544	252841166
7	51.37	chrVII:18353106	244222839
7	51.44	chrVII:17995892	120258458
7	52.04	chrVII:20883742	252841067
7	57.33	chrVII:22842571	252841120
7	60.09	chrVII:23463111	252841116
7	67.21	chrVII:24610097	252841098
7	83.34	chrVII:26448674	252841125
7	95.8	chrVII:27757015	252841145
8	0	chrVIII:1293153	252841114
8	1.81	chrVIII:1929053	244222843
8	12.55	chrVIII:3987295	120258464
8	13.85	chrVIII:4503012	244222845
8	17.51	chrVIII:9763365	252841173
8	17.51	chrVIII:9768150	252841162
8	17.51	chrVIII:9849962	252841092
8	19.22	chrVIII:12472630	252841158
8	20.8	chrVIII:13577518	252841097
8	20.78	chrVIII:13613729	244222847
8	21.19	chrVIII:3455699	252841156
8	23.9	chrVIII:14472465	244222848
8	52.4	chrVIII:17359071	252841141
9	0	chrIX:5403530	120258474
9	4.07	chrIX:6126845	252841056

9	14.89	chrIX:8586014	244223070
9	15.36	chrIX:8851078	252841178
9	15.36	chrIX:8852807	244223071
9	20.03	chrIX:12982163	244222866
9	20.03	chrIX:13553866	252841127
9	20.04	chrIX:12869521	252841147
9	32.19	chrIX:2251670	252841064
9	54.63	chrIX:803523	252841065
10	0	chrX:5784504	252841138
10	1.08	chrX:7113953	120258483
10	4.04	chrX:8703061	120258485
10	4.45	chrX:8877592	252841109
10	5.2	chrX:9396721	252841115
10	5.82	chrX:9829411	252841153
10	6.18	chrX:10415917	252841095
10	8.98	chrX:11139448	252841128
10	9.92	chrX:11252137	244222875
10	50.14	chrX:14265366	120258486
10	50.58	chrX:14456479	252841100
10	50.58	chrX:14549101	252841122
10	50.58	chrX:15290785	252841137
11	0	chrXI:5472842	244223076
11	0	chrXI:5652180	244223077
11	0	chrXI:5653380	244223079
11	0	chrXI:5708414	244222881
11	0	chrXI:5845597	244223081
11	0	chrXI:5845760	252841177
11	5.09	chrXI:9039275	252841094
12	0	chrXII:548804	252841119
12	23.19	chrXII:1969537	252841070
12	24.1	chrXII:2181073	252841129
12	29.21	chrXII:13151981	244223083
12	29.44	chrXII:13151755	244223082
12	29.44	chrXII:14223760	244222895
12	29.51	chrXII:14346080	244223084
12	29.51	chrXII:14353450	244222896
12	32.23	chrXII:10243906	252841053
12	32.62	chrXII:10246245	252841052
12	39.82	chrXII:6399147	252841133

13	0	chrXIII:1001571	120258503
13	11.03	chrXIII:2523163	120258505
13	28.29	chrXIII:12083700	252841126
13	31.84	chrXIII:14401483	252841160
13	33.87	chrXIII:15462116	244222903
13	47.09	chrXIII:17896505	252841131
13	57.72	chrXIII:18470329	252841124
13	62.68	chrXIII:19311265	252841123
13	73.66	chrXIII:19824333	252841080
13	74.12	chrXIII:19693259	244222906
14a	0	chrXIV:10399121	244222910
14a	0.43	chrXIV:11360680	252841142
14a	1.95	chrXIV:14049917	252841090
14b	0	chrXIV:265589	252841057
14b	0	chrUn:36334731	244223000
14b	0.46	chrUn:38561237	252841055
14b	2.7	chrXIV:451065	120258511
15	0	chrXV:7198078	252841171
15	0	chrXV:7200442	244223089
15	25.15	chrXV:14693722	244222917
16	0	chrXVI:2483136	252841051
16	16.39	chrXVI:5562355	244222924
16	25.61	chrXVI:12111717	120258526
16	34.81	chrXVI:14550048	252841073
16	35.94	chrXVI:14688989	252841047
16	49.97	chrXVI:16058672	252841101
16	56.79	chrXVI:17506151	252841081
16	57.48	chrXVI:17347559	244223091
16	63.94	chrUn:26389255	244222992
16	66.27	chrUn:17922401	120258570
17	0	chrXVII:3906379	244222942
17	5.33	chrUn:2632376	252841074
17	5.33	chrUn:2776586	120258568
17	15.19	chrXVII:11037958	252841072
17	28.53	chrXVII:12528572	252841151
17	28.98	chrXVII:12599208	252841154
17	33.18	chrXVII:13079654	252841130
17	34.93	chrXVII:13481178	120258537
17	36.82	chrXVII:13795831	252841087

18	0	chrXVIII:4836241	120258539
18	0	chrXVIII:5765162	120258540
18	9.16	chrXVIII:9955470	244222951
18	12.95	chrXVIII:11086837	120258542
18	19.35	chrXVIII:12273872	252841150
18	19.4	chrXVIII:12501504	244222956
18	35.62	chrXVIII:14415132	120258548
19	0	chrXIX:583166	252841105
19	0.87	chrXIX:646137	252841146
19	4.84	chrXIX:728155	120258550
19	40.33	chrXIX:2524840	252841167
19	40.33	chrXIX:2459466	244235762
19	40.33	chrXIX:2464070	244235760
19	40.33	chrXIX:2467932	244235759
19	40.33	chrXIX:2477173	244235761
19	40.44	chrXIX:2526286	244223087
19	40.51	chrXIX:2550417	252841164
19	44.69	chrXIX:18043409	252841059
19	47.63	chrXIX:14796728	244223085
19	47.86	chrXIX:15207098	252841144
19	47.86	chrXIX:14798132	244223086
19	47.99	chrXIX:14799088	252841180
19	48.72	chrXIX:13466609	244222961
19	51.64	chrXIX:10552047	252841043
19	53.45	chrXIX:7848104	252841069
20	0	chrXX:3608355	244223093
20	11.51	chrXX:8918466	244222964
20	11.52	chrXX:8905625	244222963
20	13.51	chrXX:12436776	252841049
20	13.73	chrXX:12810044	252841048
20	15.32	chrXX:13893619	252841139
20	15.53	chrXX:14411783	252841159
20	24.43	chrXX:16253512	252841060
20	31.8	chrXX:16912820	252841161
20	31.85	chrXX:16910805	252841165
20	40.2	chrXX:17494242	252841140
20	58.45	chrXX:232763	244223092
21	0	chrUn:5488093	244222984
21	3.42	chrUn:6889743	244222988

21	3.84	chrUn:7381868	252841045
21	4.06	chrXXI:774193	252841169
21	5.4	chrXXI:1893294	252841157
21	8.78	chrXXI:5716516	252841174
21	8.78	chrXXI:5793103	244222975
21	8.78	chrXXI:7002178	244222977
21	9.22	chrXXI:7904439	252841107
21	23.01	chrXXI:10007883	120258564
21	23.65	chrXXI:10156751	252841143
21	37.51	chrXXI:11060209	120258566
21	56.07	chrXXI:11414383	120258567

For each SNP marker, the linkage group (LG) and map position in the linkage group in centimorgans (cM) is shown. Marker names are based on the position (chromosome and position in basepairs) of the SNP marker in the initial stickleback genome assembly (Broad S1, Feb 2006). For SNP markers identified from unassembled regions of the genome (chrUN), the position in basepairs is based on the composition chrUN in the UCSC genome browser. SNP marker information can be obtained using the NCBI assay ID (ss#) at http://www.ncbi.nlm.nih.gov/projects/SNP/

Table S2   Microsatellite markers used for QTL mapping

Linkage group (LG)	Map position (cM)	Marker name (chromosome:position)	Forward primer sequence	Reverse primer sequence
21	7.14	chrXXI:4004587	5'-ACCTGGGAGCAATTATGTCC-3'	5'-GCTCTGGTAATTGATGTGTTCC-3'
21	7.31	chrXXI:4500405	5'-CTTTGAGGTGATGCTTGTGC-3’	5'-ATCTTGGCCTTCTTTCAACC-3'
21	13.43	chrXXI:8545029	5'-AAGTGCATTCAGGGTTCAGC-3'	5'-TGGATTTGTCACTTGGATGG-3'
21	17.19	chrXXI:9027839	5'-TCACGTTCTCCATGAAGTGG-3'	5'-TCAATGGGTAAGAGGGATGG-3'
21	20.17	chrXXI:9481372	5'-GGGAGAACTTTCCGTGTTCG-3'	5'-GCTGCAGTTCTTTGTTCTGC-3'

For each microsatellite marker, the linkage group (LG) and map position in the linkage group in centimorgans (cM) is shown. Marker names are based on the position (chromosome and position in basepairs) of the microsatellite marker in the initial stickleback genome assembly (Broad S1, Feb 2006).
