# Supplementary material for: Genetic Architecture of Variation in the Lateral Line Sensory System of Threespine Sticklebacks
Source: G3 (Bethesda). 2012 Sep 1;2(9):1047–56. doi: 10.1534/g3.112.003079 (PMC3429919; doi:10.1534/g3.112.003079)
Supplement: Supporting Information [file supp_2.9.1047_TableS2.pdf]

Table S2 Microsatellite markers used for QTL mapping

Linkage group (LG)	Map position (cM)	Marker name (chromosome:position)	Forward primer sequence	Reverse primer sequence
21	7.14	chrXXI:4004587	5'-ACCTGGGAGCAATTATGTCC-3'	5'-GCTCTGGTAATTGATGTGTTCC-3'
21	7.31	chrXXI:4500405	5'-CTTTGAGGTGATGCTTGTGC-3’	5'-ATCTTGGCCTTCTTTCAACC-3'
21	13.43	chrXXI:8545029	5'-AAGTGCATTCAGGGTTCAGC-3'	5'-TGGATTTGTCACTTGGATGG-3'
21	17.19	chrXXI:9027839	5'-TCACGTTCTCCATGAAGTGG-3'	5'-TCAATGGGTAAGAGGGATGG-3'
21	20.17	chrXXI:9481372	5'-GGGAGAACTTTCCGTGTTCG-3'	5'-GCTGCAGTTCTTTGTTCTGC-3'

For each microsatellite marker, the linkage group (LG) and map position in the linkage group in centimorgans (cM) is shown. Marker names are based on the position (chromosome and position in basepairs) of the microsatellite marker in the initial stickleback genome assembly (Broad S1, Feb 2006).
